# An Improved Fuzzy *c*-Means Clustering Algorithm Based on Shadowed Sets and PSO

**DOI:** 10.1155/2014/368628

**Published:** 2014-11-12

**Authors:** Jian Zhang, Ling Shen

**Affiliations:** ^1^School of Mechanical Engineering, Tongji University, Shanghai 200092, China; ^2^Precision Medical Device Department, University of Shanghai for Science and Technology, Shanghai 200093, China

## Abstract

To organize the wide variety of data sets automatically and acquire accurate classification, this paper presents a modified fuzzy *c*-means algorithm (SP-FCM) based on particle swarm optimization (PSO) and shadowed sets to perform feature clustering. SP-FCM introduces the global search property of PSO to deal with the problem of premature convergence of conventional fuzzy clustering, utilizes vagueness balance property of shadowed sets to handle overlapping among clusters, and models uncertainty in class boundaries. This new method uses Xie-Beni index as cluster validity and automatically finds the optimal cluster number within a specific range with cluster partitions that provide compact and well-separated clusters. Experiments show that the proposed approach significantly improves the clustering effect.

## 1. Introduction

Clustering is the process of assigning a homogeneous group of objects into subsets called clusters, so that objects in each cluster are more similar to each other than objects from different clusters based on the values of their attributes [1]. Clustering techniques have been studied extensively in data mining [2], pattern recognition [3], and machine learning [4].

Clustering algorithms can be generally grouped into two main classes, namely, supervised clustering and unsupervised clustering where the parameters of classifier are optimized. Many unsupervised clustering algorithms have been developed. One such algorithm is *k*-means, which assigns *n* objects to *k* clusters by minimizing the sum of squared Euclidean distance between the objects in each cluster to the cluster center. The main drawback of the *k*-means algorithm is that the result is sensitive to the selection of initial cluster centroids and may converge to local optima [5].

For handling those random distribution data sets, soft computing has been introduced in clustering [6], which exploits the tolerance for imprecision and uncertainty in order to achieve tractability and robustness. Fuzzy sets and rough sets have been incorporated in the *c*-means framework to develop the fuzzy *c*-means (FCM) [7] and rough *c*-means (RCM) [8] algorithms.

Fuzzy algorithms can assign data object partially to multiple clusters and handle overlapping partitions. The degree of membership in the fuzzy clusters depends on the closeness of the data object to the cluster centers. The most popular fuzzy clustering algorithm is FCM which is introduced by Bezdek [9] and now it is widely used. FCM is an effective algorithm, but the random selection in center points makes iterative process fall into the saddle points or local optimal solution easily. Furthermore, if the data sets contain severe noise points or if the data sets are high dimensional, such as bioinformatics [10], the alternating optimization often fails to find the global optimum. In these cases, the probability of finding the global optimum can be increased by stochastic methods such as evolutionary or swarm-based methods. Bezdek and Hathaway [11] optimized the hard *c*-means (HCM) model with a genetic algorithm. Runkler [12] introduced an ant colony optimization algorithm which explicitly minimizes the HCM and FCM cluster models. Al-Sultan and Selim [13] proposed the simulated annealing algorithm (SA) to overcome some of these limits and got promising results.

PSO is a population based optimization tool developed by Eberhart and Kennedy [14], which can be implemented and applied easily to solve various function optimization problems. Runkler and Katz [15] introduced two new methods for minimizing the reformulated objective functions of the FCM clustering model by PSO: PSO-*V* and PSO-*U*. In order to overcome the shortcomings of FCM, a PSO-based fuzzy clustering algorithm was discussed [16]; this algorithm uses the global search capacity of PSO to overcome the shortcomings of FCM. For finding more appropriate cluster centers, a generalized FCM optimized by PSO algorithm [17] was proposed.

Shadowed sets are considered as a conceptual and algorithmic bridge between rough sets and fuzzy sets, thereby incorporate the generic merits, and have been successfully used for unsupervised learning. Shadowed sets introduce (0,1) interval to denote the belongingness of those clustering points, and the uncertainty among patterns lying in the shadowed region is efficiently handled in terms of membership. Thus, in order to disambiguate and capture the essence of a distribution, recently the concept of shadowed sets has been introduced [18], which can also raise the efficiency in the iteration process of the new prototypes by eliminating some “bad points” that have bad influence on cluster structure [19, 20]. Compared with FCM, the capability of shadowed *c*-means is enhanced when dealing with outlier [21].

Although lots of clustering algorithms based on FCM, PSO, or shadowed sets were proposed, most of them need to input the preestimated cluster number *C*. To obtain the desirable cluster partitions in a given data, commonly *C* is set manually, and this is a very subjective and somewhat arbitrary process. A number of approaches have been proposed to select the appropriate *C*. Bezdek et al. [22] suggested the rule of thumb *C* ≤ *N* ^1/2^ where the upper bound must be determined based on knowledge or applications about the data. Another approach is to use a cluster validity index as a measure criterion about the data partition, such as Davies-Bouldin (DB) [23], Xie-Beni (XB) [24], and Dunn [25] indices. These indices often follow the principle that the distance between objects in the same cluster should be as small as possible and the distance between objects in different clusters should be as large as possible. They have also been used to acquire the optimal number of clusters *C* according to their maximum or minimum value.

Therefore, we wish to find the best *C* in some range, obtain cluster partitions by considering compactness and intercluster separation, and reduce the sensitivity to initial values. Here, we propose a modified algorithm named as SP-FCM which integrates the merits of PSO and interleaves shadowed sets between stabilization iterations. And it can automatically estimate the optimal cluster number with a faster initialization than our previous approach.

The structure of the paper is as follows.  Section 2 outlines all necessary prerequisites. In Section 3, a new clustering approach called SP-FCM is presented for automatically finding the optimal cluster number.  Section 4 includes the results of experiments involving UCI data sets, yeast gene expression data sets, and real data set. In Section 5, main conclusions are covered.

## 2. Related Clustering Algorithms

In this section, we briefly describe some basic concepts of FCM, PSO, shadowed sets, and XB validity index and review the PSO-based clustering method.

### 2.1. FCM

We define *X* = {*x*_1_,…, *x*_*N*_} as the universe of a clustering data set, *B* = {*β*_1_,…, *β*_*C*_} as the prototypes of *C* clusters, and *U* = [*u*_*ij*_]_*N*×*C*_ as a fuzzy partition matrix, where *u*_*ij*_ ∈ [0,1] is the membership of *x*_*i*_ in a cluster with prototype *β*_*j*_; *x*_*i*_, *β*_*j*_ ∈ *R* ^*P*^, where* P* is the data dimensionality, 1 ≤ *i* ≤ *N*, and 1 ≤ *j* ≤ *C*. The FCM algorithm is derived by minimizing the objective function [22]

(1) $$\begin{array}{c} J_{\text{FCM}}\left(U,B,X\right)=\overset{C}{\underset{j=1}{\sum }}\overset{N}{\underset{i=1}{\sum }}u_{ij}^{m}d_{ij}^{2}\left(x_{i},\beta_{j}\right), \end{array}$$

where *m* > 1.0 is the weighting exponent on each fuzzy membership and *d*_*ij*_ is the Euclidian distance from data vectors *x*_*i*_ to cluster center *β*_*j*_. And

(2) $$\begin{array}{c} \overset{C}{\underset{j=1}{\sum }}u_{ij}=1\;\forall i=1,2,\ldots ,N,\\ 0<\overset{N}{\underset{i=1}{\sum }}u_{ij}<N\;\forall j=1,2,\ldots ,C,\\ d_{ij}=\left\|x_{i}-\beta_{j}\right\|. \end{array}$$

This produces the following update equations:

(3) $$\begin{array}{c} u_{ij}={\left(\overset{C}{\underset{k=1}{\sum }}{\left(\frac{d\left(x_{i},\beta_{j}\right)}{d\left(x_{i},\beta_{k}\right)}\right)}^{2/\left(m-1\right)}\right)}^{-1}, \end{array}$$

(4) $$\begin{array}{c} \beta_{j}=\frac{\left(\sum_{i=1}^{N}{\left(u_{ij}\right)}^{m}x_{i}\right)}{\left(\sum_{i=1}^{N}{\left(u_{ij}\right)}^{m}\right)}. \end{array}$$

After computing the memberships of all the objects, the new prototypes of the clusters are calculated. The process stops when the prototypes stabilize. That is, the prototypes from the previous iteration are of close proximity to those generated in the current iteration, normally less than an error threshold.

### 2.2. PSO

PSO was originally introduced in terms of social and cognitive behavior of bird flocking and fish schooling. The potential solutions are called particles which fly through the problem space by following the current best particles. Each particle keeps track of its coordinates in the problem space which are associated with the best solution that has been achieved so far. The solution is evaluated by the fitness value, which is also stored. This value is called *p*best. Another best value that is tracked by the PSO is the best value, obtained so far by any particle in the swarm. The best value is a global best and is called *g*best. The search for the better positions follows the rule as

(5) Vt+1=wVt+c1r1pbestt−Pt+c2r2gbestt−Pt,Pt+1=Pt+Vt+1,

where *P* and *V* are position and velocity vector of particle, respectively, *w* is inertia weight, *c*_1_ and *c*_2_ are positive constants, called acceleration coefficients which control the influence of *p*best and *g*best in search process, and *r*_1_ and *r*_2_ are random values in the range [0,1]. The fitness value of each particle's position is determined by a fitness function, and PSO is usually executed with repeated application of (5) until a specified number of iterations have been exceeded or the velocity updates are close to zero over a number of iterations.

### 2.3. PSO-Based FCM

In this algorithm [26], each particle Part_*l*_ represents a cluster center vector, which is constructed as

(6) $$\begin{array}{c} \text{Par}{\text{t}}_{l}=\left(P_{l1},\ldots ,P_{lj},\ldots ,P_{lC}\right), \end{array}$$

where *l* represents the *l*th particle, *l* = 1,2,…*L*, *L* is the number of particles, and *L* < *N*. *P*_*lj*_ is the *j*th cluster center of particle Part_*l*_. Therefore, a swarm represents a number of candidates cluster center for the data vector. Each data vector belongs to a cluster according to its membership function and thus a fuzzy membership is assigned to each data vector. Each cluster has a cluster center per iteration and presents a solution which gives a vector of cluster centers. This method determines the position vector Part_l_ for every particle, updates it, and then changes the position of cluster center. And the fitness function for evaluating the generalized solutions is stated as

(7) $$\begin{array}{c} F\left(P\right)=\frac{1}{J_{\text{FCM}}}. \end{array}$$

The smaller is the *J*_FCM_, the better is the clustering effect and the higher is the fitness function *F*(*P*).

### 2.4. Shadowed Sets

Conventional uncertainty models like fuzzy sets tend to capture vagueness through membership values and associate precise numeric values of membership with vague concepts. By introducing *α*-cut [19], a fuzzy set can be converted into a classical set. Shadowed sets map each element of a given fuzzy set into 0, 1, and the unit interval [0, 1], namely, excluded, included, and uncertain, respectively.

**Figure 1:**
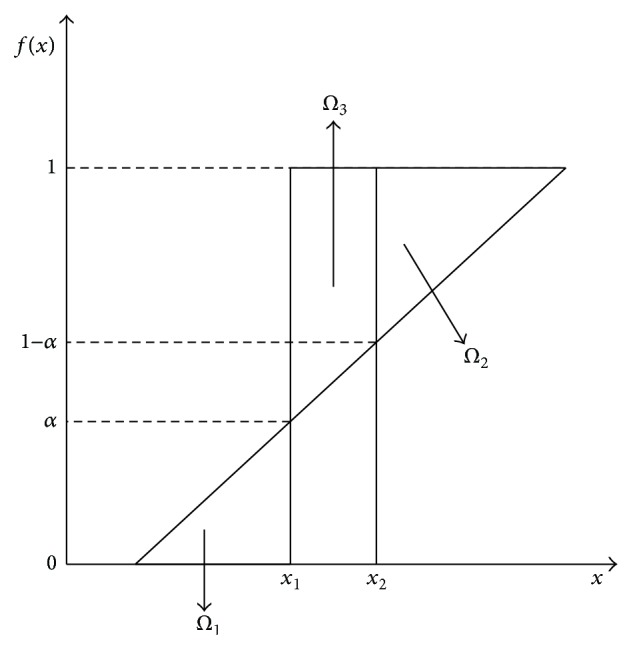
Shadowed sets induced by fuzzy function *f*(*x*).

For constructing a shadowed set, Mitra et al. [21] proposed an optimization based on balance of vagueness. As elevating membership values of some regions to 1 and at the same time reducing membership values of some regions to 0, the uncertainty in these regions can be eliminated. To keep the balance of the total uncertainty regions, it needs to compensate these changes by the construction of uncertain regions, namely, shadowed sets that absorb the previous elimination of partial membership at low and high ranges. The shadowed sets are induced by fuzzy membership function in Figure 1.

Here *x* denotes the objects; *f*(*x*)∈[0,1] is the continuous membership function of the objects belonging to a cluster. The symbol *Ω*_1_ shows the reduction of membership, the symbol *Ω*_2_ depicts the elevation of membership, and the symbol *Ω*_3_ shows the formation of shadows. In order to balance the total uncertainty, the retention of balance translates into the following dependency:

(8) $$\begin{array}{c} \Omega_{1}+\Omega_{2}=\Omega_{3}. \end{array}$$

And the integral forms are given as

(9) $$\begin{array}{c} \Omega_{1}=\int_{x:f(x)\leq \alpha }f\left(x\right)dx,\;\;\Omega_{2}=\int_{x:f(x)\geq 1-\alpha }\left(1-f\left(x\right)\right)dx,\\ \Omega_{3}=\int_{x:\alpha <f(x)<1-\alpha }dx. \end{array}$$

The threshold of reducing and elevating is *α* and 1 − *α* (*α* ∈ (0,0.5)). The optimal value of *α* should be acquired by translating it into the minimization of the following objective function:

(10) $$\begin{array}{c} O\left(\alpha \right)=\left|\Omega_{1}+\Omega_{2}-\Omega_{3}\right|. \end{array}$$

For a fuzzy set with discrete membership function, the balance equation is modified as

(11) Oαj=∑uij≤αjuij+∑uij≥ujmax⁡−αjujmax⁡−αj − carduij ∣ αj<uij<ujmax⁡−αj.

In order to find the best *α*_*j*_, it should satisfy the following optimal problem:

(12) $$\begin{array}{c} \alpha_{j}=\underset{\alpha_{j}}{\arg\;\min}\;O\left(\alpha_{j}\right), \end{array}$$

where *u*_*ij*_ ∈ [0,1] is the membership of *x*_*i*_ in a cluster with prototype *β*_*j*_; *u*_*j*_max⁡__ and *u*_*j*_min⁡__ denote the highest and lowest membership values to the *j*th cluster; and *α*_*j*_ is the threshold of the *j*th cluster. The range of feasible values of threshold *α*_*j*_ is [*u*_*j*_min⁡__, (*u*_*j*_min⁡__ + *u*_*j*_max⁡__)/2] [19].

This approach considers all membership values with respect to a fixed cluster when updating the prototype of this cluster. The main merits of shadowed sets involve the optimization mechanism for choosing separate threshold and the reduction of the burden of plain numeric computations.

### 2.5. XB Clustering Validity Index

The clustering algorithms described above require prespecification of the number of clusters. The partition results are dependent on the choice of *C*. There exist validity indices to evaluate the goodness of clustering according to a given number of clusters; therefore, these validity indices can be used to acquire the optimal value of *C* [27].

The XB index presents a fuzzy-validity criterion based on a validity function which identifies overall compact and separate fuzzy *c*-partitions. This function depends upon the data set, geometric distance measure, and distance between cluster centroids and fuzzy partition, irrespective of any fuzzy algorithm used. For evaluating the goodness of the data partition, both cluster compactness and intercluster separation should be taken into account. For the FCM algorithm with *m* = 2.0, the Xie-Beni index can be shown to be

(13) $$\begin{array}{c} S_{\text{XB}}=\frac{J_{\text{FCM}}}{Nd_{\min}^{2}}, \end{array}$$

where *d*_min⁡_ = min_*i*,*j*_‖*β*_*i*_ − *β*_*j*_‖ is the minimum distance between cluster centroids. The more separate the clusters, the larger the *d*_min⁡_ and the smaller the *S*_XB_.

## 3. Shadowed Sets-Based PSO-Fuzzy Clustering: SP-FCM

FCM strives to find *C* compact clusters in *X* where *C* is one of the specified parameters. But the process of selecting and adjusting *C* manually to obtain desirable cluster partitions in a given data set is very subjective and somewhat arbitrary. To seek the optimal cluster structure, *C* is always allowed to be overestimated [28], such that the distances between some clusters are not big enough or the membership values of some objects with different clusters are adjacent and ambiguous in a given data set. And, in this case, the modification of prototypes through long time iteration is meaningless.

The main subject of cluster validation is the evaluation of clustering results to find the partitioning that best fits the data set. Based on the foregoing algorithms, we wish to find cluster partitions that contain compact and well-separated clusters. In our algorithm *C* is also overestimated and the clusters compete for data membership. We can set [*C*_min⁡_, *C*_max⁡_] as the reasonable range of cluster number based on the knowledge of the data. This provides a more transparent and tractable process of cluster number reduction. Considering the fuzzy partition matrix *U* = [*u*_*ij*_]_*N*×*C*_, each column is comprised of the membership values of all feature vectors *x*_*i*_ with a single cluster center. Thus, an optimal threshold *α*_*j*_ (*j* = 1,2,…*C*) for each column should be found to create a harder partition by (12). The amount of data which are assigned membership value equal to 1 is identified as the cardinality of corresponding cluster. According to *α*_*j*_, the cardinality of the *j*th column is

(14) $$\begin{array}{c} M_{j}=\text{card}\left\{u_{ij}\;|\;u_{ij}\geq u_{j_{\max}}-\alpha_{j}\right\}. \end{array}$$

Here, the threshold is not subjectively user-defined but it is established on the balance of uncertainty and can be adjusted automatically in the clustering process. This property of shadowed sets can be used to reduce the cluster number. In order to control the convergence speed, the decision to delete clusters can be based on some thresholds. Different threshold values should be set for different data sets depending on the cluster structure and size of data sets. Here, a threshold *ε* and attrition rate *ρ* (0 < *ρ* < 1) are set. The decision to delete clusters in SP-FCM is based solely on cluster cardinality and the threshold  *ε*. If *ε* is too small, *C* is reduced more slowly and it may stop prematurely before the optimal cluster number is found. On the other hand, if *ε* is too large, *C* may be reduced too drastically. In our method, clusters whose cardinalities *M*_*j*_ < *ε* are considered as “candidates” for removal. And we can remove up to ⌊*ρ* × *C*⌋ clusters having the lowest cardinality from the pool of candidates specified by *ε*. Limiting the number of clusters that can be removed at one time prevents *C* from being reduced too drastically when *ε* is set too high for a given data set. This would automatically estimate the best cluster number while also utilizing a faster, consistent, and repeatable initialization technique. For evaluating the goodness of the data partition, both cluster compactness and intercluster separation should be taken into account. Hence the XB index is adopted.

For each *C* in the range of [*C*_min⁡_, *C*_max⁡_] a set of cluster validity indexes were calculated, where *C*_max⁡_ is the initial cluster number which is set to be much larger than the expected cluster number. The partition matrix with *C* clusters with the best aggregate validity index is selected as the final cluster partition. The SP-FCM algorithm is summarized as in Algorithm 1.

Here, if ⌊*ρ* × *C*⌋ is equal to 0, we can let it to be 1. This means that the cluster with the lowest cardinality may be removed. The initial *C*_max⁡_ cluster prototypes can be initialized using exemplars from data points selected by *β*_*j*_ = *x*_⌊(*N*/*C*_max⁡_)*j*⌋_. After termination, the *B* and *U* from *C* ∈ [*C*_min⁡_, *C*_max⁡_] with the best cluster validity index *S*_XB_ are selected as the final cluster prototype and partition.

## 4. Experimental Results 

In this section, the performance of FCM, RCM, shadowed *c*-means (SCM) [21], shadowed rough *c*-means (SRCM) [19], and SP-FCM algorithms is presented on four UCI datasets, four yeast gene expression datasets, and real data. For evaluating the convergence effect, the fundamental criterion can be described as follows: the distance between different objects in the same cluster should be as close as possible; the distance between different objects in different cluster should be as far as possible. Here we use DB index and Dunn index to evaluate the clustering effect. For a given data set and *C* value, the higher the similarity values within the clusters and the intercluster separation, the lower the DB index value. A good clustering procedure should make the value of DB index as low as possible. Reversely, higher values of the Dunn index indicate better clustering in the sense that the clusters are well separated and relatively compact. The details of experiments are mentioned below.

### 4.1. UCI Data Set

In our experiments, totally four UCI data sets are used, including 4-dimensional Iris, 13-dimensional Wine, 10-dimensional Glass, and 34-dimensional Ionosphere. There are 3 clusters in data set of Iris, each of which has 50 data patterns; 3 clusters in data set of Wine, which have 50, 60, and 68 data patterns; 6 clusters in data set of Glass, which have 30, 35, 40, 42, 36, and 31 separately; and 2 clusters in data set of Ionosphere, which have 226 and 125 data patterns. The validity indices of each method are compared in Table 1. SP-FCM can identify compact groups compared to other algorithms when given the cluster number *C*. It can also be seen that SRCM and SP-FCM have more obvious advantages than FCM, RCM, and SCM. SP-FCM performs slightly better than SRCM in most cases due to the global search ability which enables it to converge to an optimum or near optimum solutions. Moreover, shadowed set- and rough set-based clustering methods, namely, SP-FCM, SRCM, RCM, and SCM, perform better than FCM. It implies that the partition of approximation regions can reveal the nature of data structure and only the lower bound and boundary region of each cluster have positive contribution in the process of updating the prototypes.

**Figure 2:**
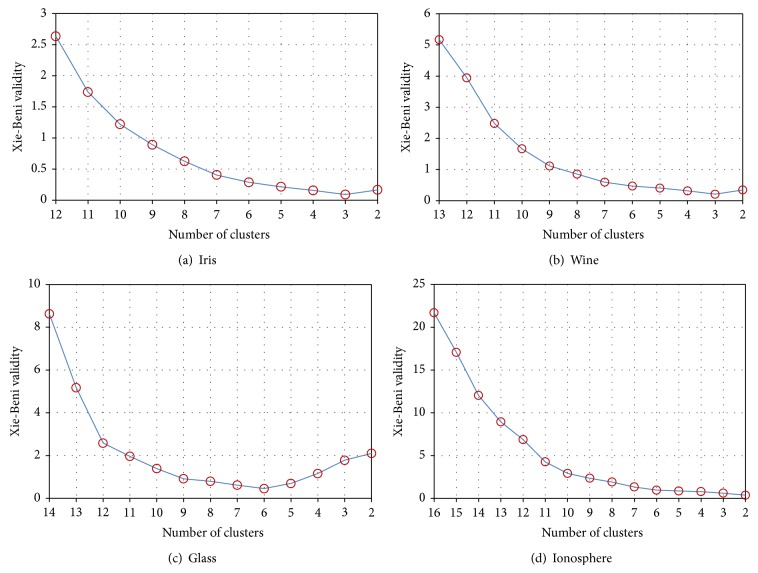
XB validity index of four UCI data sets with cluster number* C*.

As usual, the number of clusters is implied by the nature of the problem. Here, with the shadowed sets involved, one can anticipate that the optimal number of clusters could be found. The fuzzification coefficient *m* can be optimized; however, it is common to assume a fixed value of 2.0, which associates with the form of the membership functions of the generated clusters. For testing the SP-FCM algorithm, the rule *C* ≤ *N* ^1/2^ is adopted, and the range of the expected cluster number can be set as (1) Iris, [*C*_min⁡_ = 2, *C*_max⁡_ = 12]; (2) Wine, [*C*_min⁡_ = 2, *C*_max⁡_ = 13]; (3) Glass, [*C*_min⁡_ = 2, *C*_max⁡_ = 14]; (4) Ionosphere, [*C*_min⁡_ = 2, *C*_max⁡_ = 16]. The swarm size is set as *L* = 20, the maximum iteration number of PSO *T* = 50, and, for cluster reduction, the cluster cardinality threshold *ε* = 10 and the attrition rate *ρ* = 0.1. In each cycle, we get the distribution of every cluster, remove part of them according to their cardinality, and calculate the XB index, and the cluster number *C* varies from *C*_max⁡_ to *C*_min⁡_. After ending the circulation, the partition with the lowest value is selected as the final result.  Figure 2 presents the validity indices in the process of generating the optimal cluster number. Smaller values indicate more compact and well-separated clusters. The validity indices reach their minimum value at *C* = 3, 3, 6, and 2 separately, which correspond to the final cluster prototype and the best partition. Through the shadowed sets and PSO approaches, the influence of each boundary region to the formation of the prototypes and the clusters can be properly resolved. Although more computing time is required to run SP-FCM, the reasonable result can be acquired for processing the overlapping and vagueness data patterns.

### 4.2. Yeast Gene Expression Data Set

There are four yeast gene expression data sets used in the experiments, including GDS608, GDS2003, GDS2267, and GDS2712 downloaded from Gene Expression Omnibus. The number of classes and samples of GDS608 is 26 and 6303; for GDS2003, the number of classes and samples is 23 and 5617, for GDS2267 is 14 and 9275, and for GDS2712 is 15 and 9275.  Table 2 presents the validity indices of different methods after the cluster number *C* was given. The SP-FCM and SRCM obtain the same effect and perform better than other clustering algorithms. The improvement can be attributed to the fact that the global search capacity of PSO is conducive to finding more appropriate cluster centers while escaping from local optima.

**Figure 3:**
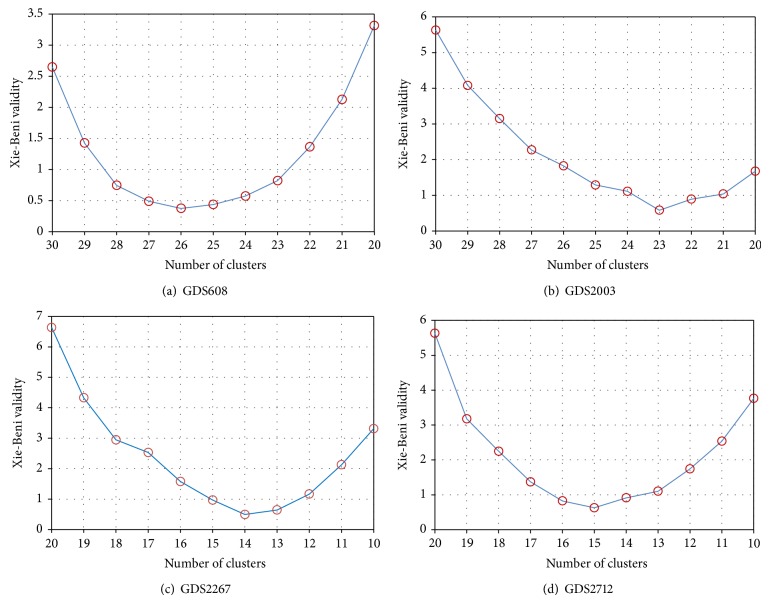
XB validity index of four yeast gene expression data sets with cluster number *C*.

For getting the optimum *C* automatically, we let *m* = 2.0, *c*_1_ = 1.49, *c*_2_ = 1.49, and *w* = 0.72, and the rule *C* ≤ *N* ^1/2^ is adopted. The swarm size is set as *L* = 20, the maximum iteration number of PSO is *T* = 80, and, for cluster reduction, the range of the expected cluster number, the cluster cardinality threshold *ε*, and the attrition rate *ρ* can be set as (1) GDS608, [*C*_min⁡_ = 20, *C*_max⁡_ = 80], *ε* = 20, *ρ* = 0.05; (2) GDS2003, [*C*_min⁡_ = 20, *C*_max⁡_ = 75], *ε* = 20, *ρ* = 0.05; (3) GDS2267, [*C*_min⁡_ = 10, *C*_max⁡_ = 96], *ε* = 20, *ρ* = 0.08; (4) GDS2712, [*C*_min⁡_ = 10, *C*_max⁡_ = 96], *ε* = 20, *ρ* = 0.08. In each cycle, we get the distribution of every cluster, remove part of them according to their cardinality, and calculate the XB index, and the cluster number *C* varies from *C*_max⁡_ to *C*_min⁡_. The partition with the lowest value is selected as the final result after the loop is ended. As seen in Figure 3, for GDS608, at the beginning the cluster number decreases at a faster rate, it takes 26 iterations to reduce the cluster number from *C* = 80 to *C* = 30 and 4 iterations from *C* = 30 to *C* = 26, and the XB index begins to increase when the cluster number *C* < 26. For GDS2003, it takes 24 iterations to reduce the cluster number from *C* = 75 to *C* = 30 and 7 iterations from *C* = 30 to *C* = 23, and the XB index begins to increase when the cluster number *C* < 23. For GDS2267, it takes 23 iterations to reduce the cluster number from *C* = 96 to *C* = 20 and 6 iterations from *C* = 20 to *C* = 14, and the XB index begins to increase when the cluster number *C* < 14. For GDS2712, it takes 23 iterations to reduce the cluster number from *C* = 96 to *C* = 20 and 5 iterations from *C* = 20 to *C* = 15, and the XB index begins to increase when the cluster number *C* < 15. Here, the advantages of fuzzy sets, PSO, and shadowed sets are integrated in the SP-FCM and make this algorithm applicable to deal with overlapping partitions, the uncertainty, and vagueness arising from the boundary regions, and the optimization process in the shadowed sets makes this method robust to outliers, so that the approximation regions of each cluster can be determined accurately and the obtained prototypes approach the desired locations.

### 4.3. Real Data

**Figure 4:**
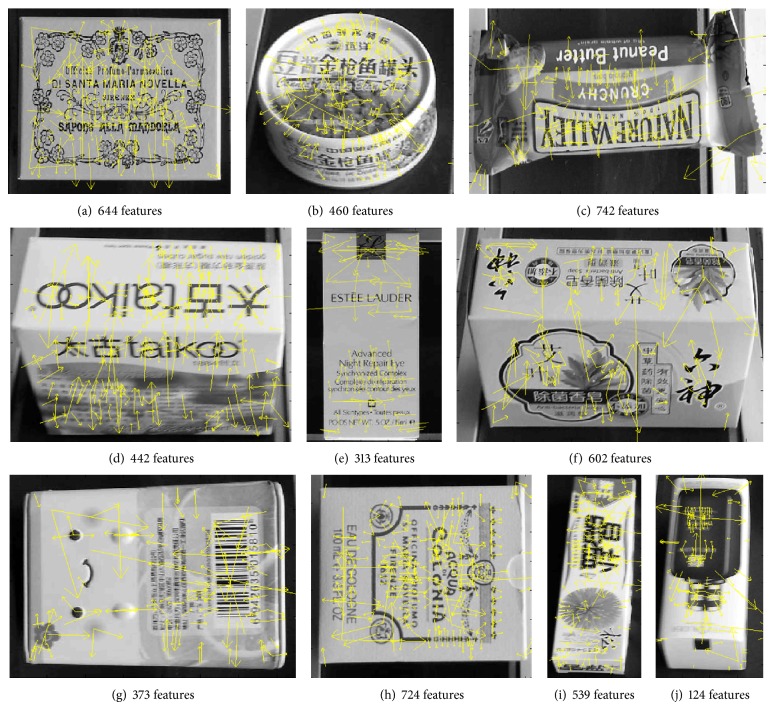
Ten package images with SIFT features.

**Figure 5:**
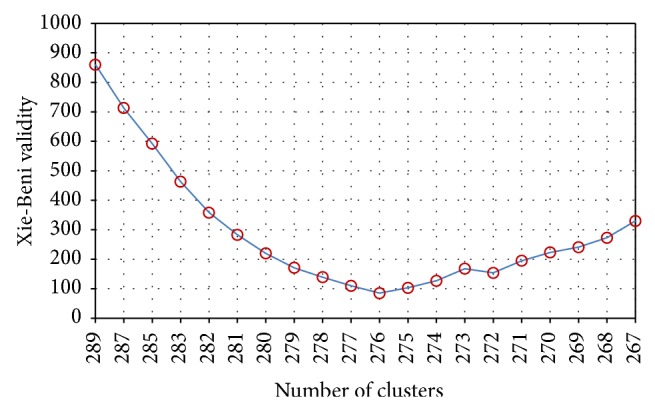
XB validity index of bag data set with cluster number *C*.

In this experiment totally 10 different packages are tested. Each package is represented by 100 frames captured from different angles by camera, and each frame is extracted SIFT feature points which are used for training a recognition system.  Figure 4 shows some images with their SIFT keypoints. And this data set is comprised of 248150 descriptors. We let *m* = 2.0, *c*_1_ = 1.49, *c*_2_ = 1.49, *w* = 0.72, *L* = 20, *ε* = 30, and *ρ* = 0.01 for the SP-FCM and choose the reasonable range [*C*_min⁡_ = 200, *C*_max⁡_ = 360] according to the category amount of packages and distribution of keypoints in each image. Eighty iterations of PSO are run on each given *C* to produce the cluster prototype *B* and partition matrix *U* as the starting point for the shadowed sets. Longer PSO stabilization is needed to obtain more stable cluster partitions. Within each cluster, the optimal *α*_*j*_ decides the cardinality and realizes cluster reduction, and XB index is calculated. Each *C*-partition is ranked using this index and selected as the final output by the smallest index value that indicates the best compact and well-separated clusters. At the beginning, the cluster number decreases at a faster speed; it takes 26 iterations to reduce the cluster number from *C* = 360 to *C* = 289 and 20 iterations from *C* = 289 to *C* = 267. The XB index increases at a relatively faster rate when the cluster number *C* < 267.  Figure 5 shows the XB index for *C* ∈ [267, 289]. The index reaches its minimum value at *C* = 276 that means the best partition for this data set is 276 clusters.  Table 3 exhibits the comparative analysis of convergence effect. As expected, SP-FCM can provide sound results for the real data; the performance is assessed by those validity indices.

## 5. Conclusions

This paper presents a modified fuzzy *c*-means algorithm based on the particle swarm optimization and shadowed sets to perform unsupervised feature clustering. This algorithm called SP-FCM utilizes the global search property of PSO and vagueness balance property of shadowed sets, such that it can estimate the optimal cluster number as it runs through its alternating optimization process. SP-FCM as a randomized based approach has the capability to alleviate the problems faced by FCM, which has some demerits of initialization and falling in local minima. Moreover, this algorithm avoids the subjective and somewhat arbitrary trials to estimate the appropriate value of cluster number, and it enhances this capability to find the optimal cluster number within a specific range using cluster validity measures as indicators. The use of XB validity index allows the algorithm to find the optimum cluster number with cluster partitions that provide compact and well-separated clusters. From the experiments, we have shown that the SP-FCM algorithm produces good results with reference to DB and Dunn indices, especially to the high dimension and large data cases.

## Figures and Tables

**Algorithm 1:**
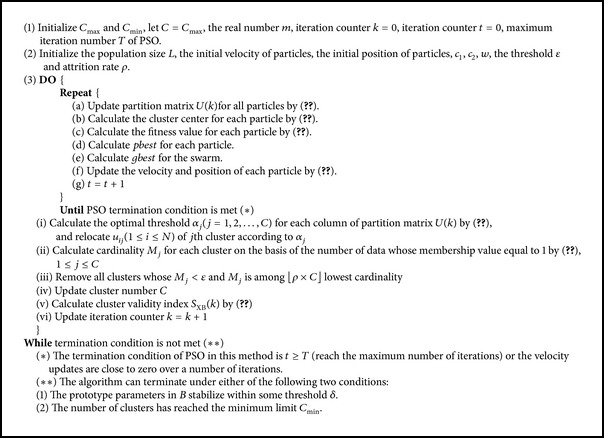
SP-FCM.

**Table 1:** Performance of FCM, RCM, SCM, SRCM, and SP-FCM on four UCI data sets.

Different indices	Algorithm	Data sets			
		Iris	Wine	Glass	Ionosphere
DB index	FCM	0.7642	0.8803	2.2971	2.0587
	RCM	0.6875	0.5692	1.9635	1.5434
	SCM	0.6862	0.5327	1.8495	1.4763
	SRCM	0.6613	0.4436	1.5804	1.3971
	SP-FCM	0.6574	0.4328	1.5237	1.4066

Dunn index	FCM	2.3106	2.5834	0.1142	0.8381
	RCM	2.7119	2.8157	0.2637	1.0233
	SCM	2.4801	2.7992	0.3150	1.0319
	SRCM	3.0874	3.1342	0.5108	1.1924
	SP-FCM	3.3254	3.1764	0.4921	1.2605

**Table 2:** Performance of FCM, RCM, SCM, SRCM, and SP-FCM on four yeast expression data sets.

Different indices	Algorithm	Data sets			
		GDS608	GDS2003	GDS2267	GDS2712
DB index	FCM	2.0861	2.4671	1.5916	1.9526
	RCM	1.6109	2.2104	1.0274	1.2058
	SCM	1.5938	2.1346	0.8946	1.0965
	SRCM	1.3274	1.9523	0.7438	0.7326
	SP-FCM	1.2958	1.8946	0.7962	0.6843

Dunn index	FCM	0.2647	0.2976	0.4208	0.3519
	RCM	0.3789	0.3981	0.7164	0.6074
	SCM	0.3865	0.3775	0.8439	0.6207
	SRCM	0.5126	0.4953	0.9759	0.8113
	SP-FCM	0.5407	0.5026	0.9182	0.8049

**Table 3:** Performance of FCM, RCM, SCM, SRCM, and SP-FCM on package datasets.

Different indices	Algorithms				
	FCM	RCM	SCM	SRCM	SP-FCM
DB index	184.569	159.671	143.194	124.038	107.826
Dunn index	92.647	116.298	125.376	169.422	167.313
